# Laissez-Faire Leadership and Affective Commitment: the Roles of Leader-Member Exchange and Subordinate Relational Self-concept

**DOI:** 10.1007/s10869-020-09700-9

**Published:** 2020-06-26

**Authors:** Véronique Robert, Christian Vandenberghe

**Affiliations:** HEC Montréal, 3000 chemin Côte Ste-Catherine, Montréal, Québec H3T2A7 Canada

**Keywords:** Laissez-faire leadership, Leader-member exchange, Affective organizational commitment, Relational self-concept, Identity orientation framework, Social exchange theory

## Abstract

Although the detrimental effects of laissez-faire leadership are well documented, research on the underlying mechanisms and the boundary conditions associated with these effects remains scarce. Using the identity orientation framework and social exchange theory, we propose that employees with stronger relational self-concepts are more likely to be affected by laissez-faire leadership. As these employees define themselves through dyadic relationships, they may react more negatively to laissez-faire leadership by diminishing their contributions to mutual goals and reducing their affective organizational commitment. These predictions were tested within a three-wave longitudinal study through structural equations modeling analyses with full information maximum likelihood estimation on a sample of employees from multiple organizations (*N* = 449). As predicted, the relational self-concept was associated with a stronger negative effect of laissez-faire leadership on the contribution dimension of leader-member exchange and a stronger negative indirect effect on affective organizational commitment. The implications of these findings for our understanding of the mechanisms related to laissez-faire leadership are discussed.

Leadership has always been at the forefront of organizational research. Most research has focused on what constitutes a good leader, neglecting negative forms of leadership (Tepper, 2000, 2007; Schyns & Schilling, 2013; Most research has focused on what constitutesMost research has focused on what constitutesZellars, Tepper, & Duffy, 2002). However, according to the principle that “bad is stronger than good” (Baumeister, Bratslavsky, Finkenauer, & Vohs, 2001), negative forms of leadership may be more influential than positive forms of leadership. It is thus surprising that this area of research has been underinvestigated (Hinkin & Schriesheim, 2008a; Judge & Piccolo, 2004). Despite recent interest into destructive leadership (Schyns & Schilling, 2013), more passive yet destructive forms of leadership such as laissez-faire leadership did not receive the same attention (Che, Zhou, Kessler, & Spector, 2017). Passive forms of leadership, which include laissez-faire as the most extreme passivity of leaders, can still have detrimental effects on employees and organizations (Hinkin & Schriesheim, 2008a; Kelloway, Sivanathan, Francis, & Barling, 2005; Skogstad, Einarsen, Torsheim, Aasland, & Hetland, 2007). For example, laissez-faire leadership was found to be associated with reduced job satisfaction, leader effectiveness, satisfaction with the leader (Judge & Piccolo, 2004), and performance (Yammarino, Spangler, & Bass, 1993). Similarly, a study (Skogstad et al., 2014) found laissez-faire leadership to be the sole (negative) leadership predictor of job satisfaction over a 2-year period. However, despite being one of the most prevalent forms of negative leadership in modern organizations (Aasland, Skogstad, Notelaers, Nielsen, & Einarsen, 2010), laissez-faire leadership has been understudied (Skogstad, Hetland, Glasø, & Einarsen, 2014). Organizational research would gain from investigating this particular type of (negative) leadership given both its prevalence and its likely detrimental effects on employees and organizations.

Laissez-faire leadership is part of the *full-range leadership model* (Avolio, 2011), one of the most established (Den Hartog, Van Muijen, & Koopman, 1997) and popular models of leadership (Dumdum, Lowe, & Avolio, 2002; Judge & Piccolo, 2004; Lowe, Kroeck, & Sivasubramaniam, 1996), which also comprises transformational and transactional dimensions. Defined as avoidance and abdication of one’s responsibilities (Hinkin & Schriesheim, 2008b; Skogstad, Hetland, et al., 2014), “laissez-faire has been consistently found to be the least satisfying and least effective management style” (Bass & Bass, 2008, p. 145). However, as research has mainly focused on the direct effects of laissez-faire leadership (Bass & Bass, 2008; Hinkin & Schriesheim, 2008a), the mechanisms and contextual boundaries associated with these effects have received little attention, which is a gap we intend to fill with the current study. Our attempt at doing so resonates with the call for a more nuanced approach to laissez-faire leadership (Wong & Giessner, 2018), as its effects may depend on the context (Yang, 2015; Yang & Li, 2017). By shedding light on these processes, we take a step toward understanding how the detrimental effects of laissez-faire leadership can be reduced, hence providing clues for practitioners.

First, laissez-faire leadership may differentially affect individuals depending on their individual dispositions. An important individual disposition that has been considered in prior leadership research is the self-concept (Lord, Brown, & Freiberg, 1999; van Knippenberg, van Knippenberg, De Cremer, & Hogg, 2004). The self-concept refers to the ways in which people define themselves and, as such, influences the perceptions of oneself and others (Brewer & Gardner, 1996; Lord & Brown, 2004; Markus & Wurf, 1987). It is composed of distinct motivations, sources of self-worth, and self-knowledge (Brickson, 2000). Multiple levels of the self-concept have been identified, namely, the individual, relational, and collective levels (e.g., Brewer & Gardner, 1996; Lord & Brown, 2004). Since leadership involves dyadic relationships between leaders and subordinates, a relational self-concept, which refers to the significance of dyadic relationships in people’s life (Johnson & Saboe, 2011), is a salient characteristic that may influence employee reactions to leaders (Brewer & Gardner, 1996). Employees with a strong relational self-concept are likely more affected by, and to react more strongly to, laissez-faire leadership because such leadership poses a threat to their goals, needs, and identity-defining relationship (Wisse & Sleebos, 2016). The absence of decisions and interactions with the leader may violate their expectations that a leader should attend to work-related problems and their relational needs (Lord & Brown, 2001). Therefore, individuals with strong relational self-concepts may experience laissez-faire leadership as disappointing, resulting in negative attitudes toward their supervisors and the organization.

Second, we explore the possibility that laissez-faire leadership may negatively affect the quality of the exchange relationship between employees and leaders. Leader-member exchange (LMX) theory suggests that leaders develop differential relationships with employees, ranging from low-quality to high-quality relationships (Boies & Howell, 2006; Chen, He, & Weng, 2018; Erdogan & Bauer, 2010; Liden & Graen, 1980). As laissez-faire leadership involves the abdication of one’s responsibilities, it may result in reduced LMX, particularly among employees with strong relational self-concepts. As these individuals are more sensitive to expressions of support and recognition and the active involvement of their leaders in decisions (Brewer & Gardner, 1996), laissez-faire leaders—because they do not attend to employees’ relational needs—will not be able to entice them to cooperate and contribute to mutual goals (De Cremer, 2003). Among the dimensions of LMX (i.e., affect, loyalty, contribution, and professional respect; Liden & Maslyn, 1998), one particularly reflects that “currency of exchange” (Dienesch & Liden, 1986; Greguras & Ford, 2006; Law, Wang, & Hui, 2010; Maslyn & Uhl-Bien, 2001) we allude to here. Specifically, the *contribution* dimension of LMX (i.e., the activity put forth toward mutual goals; Liden & Maslyn, 1998) is most likely to be affected because laissez-faire leadership involves a failure to invest in the relationship with the employee. Thus, as a result of laissez-faire leadership, employees with strong relational self-concepts may be inclined to reduce their contributions to mutual goals. We further argue that a lack of contribution by these employees will in turn lead to reduced affective organizational commitment (AOC) because it is well established that relationships with supervisors have implications for attitudes toward the organization (Dulebohn, Bommer, Liden, Brouer, & Ferris, 2012).

This study contributes to the leadership literature in several ways. First, we extend this literature by delving into the mechanisms and boundary conditions explaining how laissez-faire leadership negatively relates to AOC. Our focus is on examining the quality of the relationship between employees and leaders (i.e., LMX) as a primary reason why laissez-faire may affect AOC. Second, in doing so, we take a disaggregated approach to LMX and identify its *contribution* dimension as the most relevant aspect of LMX that should be affected by laissez-faire leadership. To further demonstrate the unique sensitivity of LMX’s contribution dimension to laissez-faire leadership, this study shows in parallel that the other LMX dimensions (i.e., affect, loyalty, and professional respect) are not affected by laissez-faire leadership. Third, we examine employees’ relational self-concepts as a boundary condition and, as such, depart from the leader-centric approaches that dominate the field (Schyns & Schilling, 2013). The relational self-concept is used as an individual difference variable that magnifies the value that individuals attribute to dyadic relationships. Fourth, our focus on laissez-faire leadership as an antecedent to LMX and AOC breaks new ground by expanding the spectrum of negative antecedents to these constructs. Finally, our hypotheses were tested within a dynamic perspective as we controlled for the baseline levels of our mediator and outcome variables in a three-wave longitudinal study. Hypotheses are developed in the next sections.

## Theoretical Framework and Hypotheses

### Laissez-Faire Leadership

Laissez-faire leadership is characterized by avoidance and inaction (Bass & Bass, 2008; Avolio, 2011; Hinkin & Schriesheim, 2008b; Skogstad, Hetland, et al., 2014). Laissez-faire leaders avoid making decisions, abdicate their responsibilities, delay actions, and refrain from using the authority associated with their roles (Bass & Bass, 2008; Den Hartog et al., 1997). They also fail to provide feedback and recognition to subordinates (Hinkin & Schriesheim, 2008b) and they tend to ignore followers’ needs, as they do not deal with work-related problems (Yukl, 2010). These leaders do not take sides in disputes and are disorganized in dealing with priorities (Bass, 1998). Based on their survey, Aasland et al. (2010) noted that 21% of employees had experienced laissez-faire behaviors from their leaders during the previous six months, making laissez-faire the most prevalent form of negative leadership.

Neglecting one’s responsibilities as a leader harms both the organization and the subordinates (Hinkin & Schriesheim, 2008a; Skogstad et al., 2007). Laissez-faire leadership is not only ineffective but also destructive (Aasland et al., 2010; Einarsen, Aasland, & Skogstad, 2007; Skogstad, Aasland, et al., 2014; Skogstad, Hetland, et al., 2014). Empirically, laissez-faire leadership has been found to be associated with reduced subordinate effort (Bass & Stogdill, 1990), performance (Yammarino et al., 1993), job satisfaction, perceived leader effectiveness, and satisfaction with the leader (Judge & Piccolo, 2004); increased stress and interpersonal conflicts (Skogstad et al., 2007); and more role ambiguity and role conflict (Skogstad et al., 2007; Skogstad, Hetland, et al., 2014). However, the inactivity characterizing laissez-faire leadership makes this style of leadership unique and distinct from other forms of negative leadership because its negative consequences result from the absence of constructive behaviors rather than from the presence of destructive ones (Kelloway, Mullen, & Francis, 2006). Therefore, further inquiry into laissez-faire leadership is warranted.

### Laissez-Faire Leadership and Leader-Member Exchange

We posit that a primary mechanism through which laissez-faire leadership may affect employees pertains to the quality of the exchange relationship with the leader or LMX (e.g., Buch, Martinsen, & Kuvaas, 2015). Indeed, employees may be unmotivated to uphold a good relationship with a leader with whom they expect to have limited interactions (van Knippenberg & Steensma, 2003). According to social exchange theory (Blau, 1964), employees invest in a relationship when they feel that contributing their time and energy may lead to reciprocal exchanges. However, laissez-faire leaders fail to provide resources such as information, challenging task assignments, and autonomy-supportive conditions. In such circumstances, employees may feel they are not receiving their due in the relationship with their leader, which may reduce their desire to engage in tasks and duties beyond what is formally required.

The exchange of resources and opportunities is central to LMX development (Liden & Graen, 1980) and depending on the resources/opportunities that are valued by the exchange partners (Graen & Cashman, 1975), different “currencies of exchange” may be salient to LMX (Dienesch & Liden, 1986; Law et al., 2010). Liden and Maslyn (1998) (see also Dienesch & Liden, 1986) developed a conceptualization of LMX comprising four dimensions reflecting different aspects of these currencies: *affect* (i.e., mutual affection based on interpersonal attraction), *loyalty* (i.e., the expression of public support for the goals and the other member’s character), *contribution* (i.e., the amount, direction and quality of work toward mutual goals), and *professional respect* (i.e., the perception of reputation and excellence). While many studies have adopted a unidimensional view of LMX (Dulebohn, Wu, & Liao, 2017), it is likely that high LMX is derived from different dimensions depending on circumstances (Liden & Maslyn, 1998; Maslyn & Uhl-Bien, 2001), such as the leadership style adopted (Lee, 2005). Thus, the very nature of laissez-faire leadership may indicate which dimension of LMX is more likely to be solicited.

As laissez-faire leadership involves unfulfilled responsibilities, these leaders set standards that lower the value of work-related exchanges (Liden & Maslyn, 1998). Therefore, the task-related behaviors of employees (Graen & Scandura, 1987; Liden & Maslyn, 1998; Maslyn & Uhl-Bien, 2005) and employees’ own efforts to develop LMX (Maslyn & Uhl-Bien, 2001) may be limited. With laissez-faire leadership, the *contribution* dimension of LMX, which refers to the “perception of the amount, direction, and quality of work-oriented activity each member puts forth toward the mutual goals (explicit or implicit) of the dyad” (Dienesch & Liden, 1986, p. 624), is likely affected (e.g., Lee, 2005). From the employee’s perspective, LMX’s contribution reflects the subordinate’s willingness to help the leader and contribute to his or her goals. Following social exchange theory (Blau, 1964), laissez-faire leaders do not encourage subordinates to contribute to mutual goals over what is included in their job descriptions as they may think they do not receive their dues (e.g., support, recognition) in the relationship with the leader. It is also likely that LMX’s contribution dimension is mostly affected in response to laissez-faire leadership because it is the only dimension that reflects the exchange from a behavioral perspective. The other dimensions (*affect*, *loyalty*, and *respect*) do not refer to the behavioral component of the exchange relationship. Laissez-faire leaders echo to this dimension by not taking actions that would signal support and recognition to subordinates. It is thus the absence of constructive behaviors (Kelloway et al., 2006) in laissez-faire leaders that makes LMX’s contribution mostly affected.

However, as theory has stipulated that because of limited resources and time, leaders differentiate among followers (Dansereau Jr., Graen, & Haga, 1975; Graen & Cashman, 1975; Graen & Scandura, 1987; Liden & Graen, 1980; Maslyn & Uhl-Bien, 2005), distinct LMX relationships are found across followers (Boies & Howell, 2006; Chen et al., 2018; Erdogan & Bauer, 2010; Henderson, Liden, Glibkowski, & Chaudhry, 2009; Herdman, Yang, & Arthur, 2017; Le Blanc & González-Romá, 2012; Liden, Erdogan, Wayne, & Sparrowe, 2006*;* Wu, Tsui, & Kinicki, 2010). Thus, while laissez-faire leadership may lend itself to poor LMX relationships, particularly in regard to its contribution dimension, there may be variability in the extent to which employees’ relationships with their leaders are impacted by laissez-faire leadership. One factor that may explain this variability relates to employees’ self-concepts (Jackson & Johnson, 2012), which we now discuss.

### Levels of the Self-concept

Leadership practices do not operate in a vacuum (Epitropaki, Kark, Mainemelis, & Lord, 2017). Rather, leaders’ behavior interacts with the characteristics of followers (Padilla, Hogan, & Kaiser, 2007). Such interactionist perspective suggests that a better understanding of leaders’ influence can be gained by accounting for followers’ expectations about leaders’ behavior. To illustrate such individual differences, research has identified the self-concept as an important background construct that guides individuals’ reactions to leaders’ behavior (Lord et al., 1999). The self-concept is a self-regulatory mechanism that drives self-esteem and organizes self-relevant knowledge (Brewer & Gardner, 1996). As a chronic representation of identity that promotes a self-definition anchored at the individual, relational, or collective level, the self-concept influences how people feel, think, and behave (Lord & Brown, 2004; Markus & Wurf, 1987). Research has shown that the levels of the self-concept influence employees’ interpretations of leaders’ behavior (Chang & Johnson, 2010; Jackson & Johnson, 2012; Lord & Brown, 2004; Lord et al., 1999; Wu et al., 2010) and influence leaders’ effectiveness (Hogg, Martin, & Weeden, 2003; Lord & Brown, 2004; Lord et al., 1999). By extension, we expect the self-concept to play a similar role regarding laissez-faire leadership.

Three levels of the self-concept have been identified (Brewer & Gardner, 1996; Brickson, 2000; Johnson, Selenta, & Lord, 2006; Lord & Brown, 2004; Lord et al., 1999; Sedikides & Brewer, 2001; Sedikides, Gaertner, & O’Mara, 2011). The collective self-concept involves the self-definition derived from belonging to groups such as organizations or teams; the relational self-concept involves a focus on dyadic relationships as a source of identity; and the individual self-concept stresses an individual’s uniqueness and self-interests (Brewer & Gardner, 1996; Lord et al., 1999; Sedikides & Brewer, 2001; van Knippenberg et al., 2004). Even though the different levels may coexist within the same person, individuals differ regarding the importance they place on each level of the self-concept (Brewer & Chen, 2007).

Although the employee self-concept has been shown to exert a moderating role on leader effectiveness, this effect has been mostly studied using the collective self-concept (Hogg, 2001; Hogg & van Knippenberg, 2003; Lord et al., 1999; Lord & Brown, 2004; van Knippenberg & Hogg, 2003). However, the relational self-concept has been largely overlooked. This is surprising because individuals are more likely to be affected by threats at the relational level than by those at the collective level of the self (Gaertner et al., 2012). Moreover, the relational identity becomes relevant when one looks at the outcomes of the leader’s actions from the perspective of the dyadic relationship (i.e., LMX; Chang & Johnson, 2010; Lord et al., 1999; Schyns & Day, 2010). As subordinates with strong relational self-concepts place a premium on dyadic exchanges (Wisse & Sleebos, 2016) and affective bonds with specific others (Brewer & Gardner, 1996), their self-worth should be particularly dependent on how their leader responds to their relational expectations.

### Moderating Role of the Relational Self-concept

Reliable role performance is rooted in how interactions between leader and subordinate unfold and whether the partners’ role expectations are fulfilled (Graen & Scandura, 1987). By abdicating the responsibilities related to their role, laissez-faire leaders violate subordinates’ role expectations (Eagly, Johannesen-Schmidt, & van Engen, 2003; Hinkin & Schriesheim, 2008a; Skogstad et al., 2007). However, the discrepancy between employees’ expectations and leaders’ behavior is likely stronger among employees with a relational self-identity because these employees are particularly sensitive to the fulfillment of role expectations (Andersen & Chen, 2002). Indeed, these employees have important relational needs, entertain affective ties with significant others (Brewer & Gardner, 1996; Flynn, 2005; Wisse & Sleebos, 2016), and expect dyadic partners to engage in behaviors that satisfy their relational expectations. Therefore, they are likely to feel frustrated if their leader does not engage in actions liable to maintain the relationship vivid and constructive.

Laissez-faire leaders may discourage employees from investing resources in LMX (Aryee, Chen, Sun, & Debrah, 2007; Xu, Huang, Lam, & Miao, 2012). Per the tenets of social exchange theory (Blau, 1964), a balance is expected between inputs and contributions in LMX relationships (Kuvaas, Buch, Dysvik, & Haerem, 2012). As laissez-faire leaders fall short of maintaining balanced relationships (e.g., they delay decisions and do not take actions when needed), employees with relational self-concepts would experience this as a threat to their identity (Brewer & Gardner, 1996; Flynn, 2005). This is so because they tend to define themselves in terms of their relations with others (Ferris, Yan, Lim, Chen, & Fatimah, 2016). Employees with a relational self-concept may thus experience their sense of self-worth as being undermined by the laissez-faire behavior of their leader (Swann Jr., Chang-Schneider, & Angulo, 2007), which would lower their motivation to cooperate with him or her (Tyler, 2003). As a result, employees with a relational self-concept may thrive to protect themselves by reducing their contribution to the relationship (Flynn, 2005). Thus, the lack of reciprocity (Herdman et al., 2017) instilled by laissez-faire behaviors would encourage these employees to reduce their contributions to the attainment of mutual goals, which represents an integral aspect of LMX (Maslyn & Uhl-Bien, 2001). In sum, these employees would fall back on formal and contractual obligations (Erdogan & Liden, 2002; Liden & Graen, 1980*;* Shore, Bommer, Rao, & Seo, 2009).

*Hypothesis 1*: The employee’s relational self-concept will moderate the relationship between laissez-faire leadership and LMX-Contribution such that this relationship will be stronger (vs. weaker) and negative when the relational self-concept is high (vs. low).

### Affective Organizational Commitment

AOC reflects an emotional attachment to and identification with one’s organization (Allen & Meyer, 1990; Meyer & Allen, 1997). It is the most impactful component of organizational commitment (Meyer & Herscovitch, 2001) and the most robust predictor of work-related behaviors (Lavelle, Rupp, & Brockner, 2007). Multiple studies have reported a positive relationship between LMX and AOC (Dulebohn et al., 2012; Gerstner & Day, 1997; Liden & Maslyn, 1998; Liden, Wayne, & Sparrowe, 2000). AOC is one the most studied outcomes of LMX (Eisenberger et al., 2010; Meyer, 2016; Wayne et al., 2009). Liden and Maslyn (1998) theorized that the contribution dimension of LMX reflects a willingness to complete tasks that go beyond one’s job description and benefit the organization as a whole. Thus, more specifically, LMX-Contribution should be positively related to AOC (Greguras & Ford, 2006; Lee, 2005; Shore & Wayne, 1993). Indeed, since leaders carry out responsibilities and make decisions on behalf of the organization, they are seen as representing the organization (Skarlicki & Folger, 1997) and as agents connecting employees to the organization (Seers & Graen, 1984). Therefore, positive exchange relationships between leaders and employees as reflected in strong LMX-Contribution should ultimately result in stronger AOC **(**Eisenberger, Aselage, Sucharski, & Jones, 2004).

As argued above, we expect a higher relational self-concept to be associated with a more negative relationship between laissez-faire leadership and LMX-Contribution. Following a social exchange account (Blau, 1964), this effect should extend to the indirect relationship between laissez-faire leadership and AOC. That is, employees with strong relational self-concepts should feel that their needs and expectations are unfulfilled when their leaders abdicate their responsibilities because dyadic relationships occupy a central place in these individuals’ self-definitions. This feeling would encourage them to reduce their contribution to mutual goals. In turn, this decreased contribution would penalize employee commitment to the organization because the relative quality of the exchange relationship with the supervisor tends to generalize to the attachment to the organization (Eisenberger, Stinglhamber, Vandenberghe, Sucharski, & Rhoades, 2002).

*Hypothesis 2*: The employee’s relational self-concept will moderate the indirect relationship between laissez-faire leadership and AOC through LMX-Contribution such that this indirect relationship will be stronger (vs. weaker) and negative when the relational self-concept is high (vs. low).

## Method

### Sample and Procedure

Data were gathered through survey questionnaires that were administered in three waves with intervals of four months. Participants were recruited through the alumni association of a French business school. Prospective participants received an email inviting them to participate in an online study of job attitudes based on three questionnaires administered over several months. They were informed of the study objectives and ensured that participation was voluntary and responses would be kept confidential. The criteria for participation were having (a) salaried employment and (b) an identifiable supervisor. To encourage participation, the respondents had the opportunity to make a $5 gift to a charity of their choice at each wave of the surveys. The questionnaires were answered in French or English. At time 1, we measured the self-concept levels, laissez-faire leadership, LMX dimensions, AOC, and demographics, among other variables. The LMX dimensions were measured again at time 2, while AOC was also measured at time 3. The baseline (i.e., time 1) levels of the mediator (i.e., LMX-Contribution) and outcome (i.e., AOC) variables were controlled for while examining the moderation effect of the relational self-concept in the relationships among laissez-faire leadership, LMX-Contribution, and AOC. This approach provided a strong test of the longitudinal moderated mediation effects (Maxwell & Cole, 2007).

Excluding careless respondents (*n* = 4) and participants who left supervisors or organizations during the study period (*n* = 60), there remained 449 respondents at time 1, 182 at time 2, and 120 at time 3 (i.e., 27% response rate). We first examined whether respondent attrition across time was randomly distributed. Specifically, we conducted a logistic regression analysis with time 1 self-concept levels, laissez-faire leadership, LMX dimensions, AOC, and demographics predicting the probability of remaining in the sample at time 3 (Goodman & Blum, 1996). The logistic regression model was nonsignificant (*χ*^2^(13) = 15.15, *ns*) and none of the predictors was significant, indicating random attrition. Because the data were missing completely at random across time, we used full information maximum likelihood (FIML) estimation within structural equations modeling (see Results section) to test hypotheses (Ployhart & Vandenberg, 2010). This estimation procedure uses all the available information from the covariance matrix (*N* = 449) and is the recommended method for dealing with missing data (Newman, 2009).

In the final sample used for analyses, age averaged 37.67 years (*SD* = 9.00), organizational tenure averaged 6.07 years (*SD* = 5.67), and tenure with the supervisor averaged 2.95 years (*SD* = 2.28). Most of the participants were women (63%), worked full-time (92%), had a graduate-level education (94%), and were employed in organizations of 1000 or more employees (56%). They worked in various industries, such as finance and insurance (15%), professional, scientific and technical services (11%), manufacturing (7%), health care and social assistance (5%), retail trade (5%), and information and cultural industries (4%).

### Measures

When needed, French versions of the English scales were created using a translation-back-translation procedure (Schaffer & Riordan, 2003). Responses were obtained on a 5-point Likert scale ranging from 1 (*strongly disagree*) to 5 (*strongly agree*), unless otherwise specified.

#### Laissez-Faire Leadership

We measured laissez-faire leadership at time 1 using a 7-item version (Hinkin & Schriesheim, 2008a, 2008b) of the laissez-faire scale from the *Multifactor Leadership Questionnaire 5X* (Bass & Avolio, 1991). A sample item is “[In the past few weeks] my immediate supervisor avoided making decisions about my work,” with response options of 1 (*strongly disagree*) to 5 (*strongly agree*). Cronbach’s alpha for this scale was .93.

#### LMX-Contribution

Participants answered the 12-item multidimensional measure of LMX (LMX-MDM) developed by Liden and Maslyn (1998) at time 1 and time 2, which contains four 3-item scales pertaining to the four LMX dimensions. The internal consistency for the 3-item LMX-Contribution scale was .79 at time 1 and .80 at time 2. A sample item is “I do work for my supervisor that goes beyond what is specified in my job description.” For exploratory purposes, we also measured the other LMX dimensions using their respective 3-item scales: affect (e.g., “I like my supervisor very much as a person”; *α* = .90 at time 1 and .91 at time 2); loyalty (e.g., “My supervisor would defend me to others in the organization if I made an honest mistake”; *α* = .91 at time 1 and .90 at time 2); and professional respect (e.g., “I admire my supervisor’s professional skills”; *α* = .94 at time 1 and .95 at time 2).

#### AOC

We measured AOC at time 1 and time 3 using an adapted version (Bentein, Vandenberg, Vandenberghe, & Stinglhamber, 2005) of Meyer, Allen, and Smith’s (1993) 6-item scale that was developed for international replications (cf., Meyer, Barak, & Vandenberghe, 1996). A typical item is “I feel emotionally attached to this organization.” The alpha coefficient for this scale was .93 at time 1 and time 3.

#### Relational Self-concept

The relational self-concept was measured at time 1 through a 5-item scale developed by Selenta and Lord (2005) and used in Johnson et al. (2006). A factor analysis of the scale items indicated that one item (“Knowing that a close other acknowledges and values the role that I play in their life makes me feel like a worthwhile person”) had a low loading on the factor (< .40) and reduced its internal consistency. Hence, we dropped that item from the scale. The remaining 4-item scale had a reliability of .71. A sample item is “If a friend was having a personal problem, I would help him/her even if it meant sacrificing my time or money.”

#### Control Variables

While testing our hypotheses and model, we controlled for the individual and collective levels of the self-concept, as other researchers have done (e.g., Johnson & Chang, 2008; Johnson et al., 2006). Indeed, as the three levels of the self-concept are generally correlated with one another (Kashima & Hardie, 2000), controlling for the individual and collective self-concepts helps avoid confounding effects. The individual (*α* = .82) and collective (*α* = .77) self-concepts were each measured at time 1 by a 5-item scale from Selenta and Lord (2005) (see also Johnson et al., 2006). Sample items include “I often compete with my friends” and “It is important to me to make a lasting contribution to groups that I belong to,” respectively.

## Results

### Confirmatory Factor Analyses

First, as a preliminary test, we used confirmatory factor analysis (CFA) through Mplus 7.31 (Muthén & Muthén, 2010) and maximum likelihood (ML) estimation to examine the dimensionality of the LMX measure at time 2. We allowed the errors of items 7 and 8 of the scale to correlate, which is recommended when there is wording similarity (Marsh et al., 2010, 2013). The four-factor model of time 2 LMX yielded a good fit (*χ*^2^(47) = 87.00, CFI = .98, TLI = .97, RMSEA = .068, SRMR = .043) and outperformed a one-factor model (**Δ***χ*^2^(6) = 599.09, *p* < .001), supporting the idea of treating LMX dimensions (e.g., LMX-Contribution) separately. Similarly, the eight-factor model including the four LMX dimensions at time 1 and time 2 yielded a good fit (*χ*^2^(212) = 558.06, CFI = .95, TLI = .93, RMSEA = .06) and outperformed a two-factor model (time 1 LMX vs. time 2 LMX) **(***χ*^2^(27) = 1717.26, *p* < .001) and a one-factor model (*χ*^2^(28) = 2124.89, *p* < .001).

Second, we tested the distinctiveness of our variables within the hypothesized eight-factor model (i.e., time 1 laissez-faire leadership, time 1 self-concept levels, time 1 LMX-Contribution, time 1 AOC, time 2 LMX-Contribution, and time 3 AOC) and compared this model with more parsimonious models using a nested sequence approach (Bentler & Bonett, 1980). The FIML method was used because it relies on all the available information from the covariance matrix (e.g., Enders, 2010; Fitzmaurice, Laird, & Ware, 2004; Graham, 2009, 2012) and is the recommended approach in longitudinal research when respondent attrition across time is random (Ployhart & Vandenberg, 2010). The errors of parallel items were allowed to correlate across time (Geiser, 2012). In addition, the errors of two pairs of items of the same constructs were allowed to correlate within time due to wording similarity (Marsh et al., 2010, 2013) (i.e., laissez-faire: items 1 and 2; individual self-concept: items 1 and 5). These specifications were incorporated in the test of the longitudinal model (Little, 2013).

The CFA results are reported in Table 1. The hypothesized eight-factor model yielded a good fit (*χ*^2^(663) = 1373.00, CFI = .92, TLI = .91, RMSEA = .05). Moreover, this model was superior to any simpler model obtained by merging specific factors (*p* < .01). Our variables were thus distinguishable. As evidence of convergent validity, in the eight-factor model, loadings were significant (*p* < .001) and sizeable (standardized factor loadings ranged from .48 to .90).

**Table 1:** Fit indices for confirmatory factor analysis models

	*χ*^2^	*df*	CFI	TLI	RMSEA	Δ*χ*^2^	Δ*df*
1. Hypothesized eight-factor solution	1373.00*	663	.92	.91	.05	–	–
2. Seven-factor solution, combining T1 AOC and T3 AOC	1653.69*	670	.88	.87	.06	280.69*	7
3. Seven-factor solution, combining T1 LMX-C and T2 LMX-C	1498.81*	670	.90	.89	.05	125.81*	7
4. Seven-factor solution, combining T1 laissez-faire leadership and T2 LMX-C	1581.26*	670	.89	.88	.06	208.25*	7
5. Seven-factor solution, combining T1 RSC and CSC	1611.18*	670	.89	.88	.06	238.18*	7
6. Seven-factor solution, combining T1 RSC and ISC	2162.32*	670	.82	.81	.07	789.32*	7
7. Seven-factor solution, combining T2 LMX-C and T3 AOC	1549.96*	670	.90	.89	.05	176.96*	7
8. Six-factor solution, combining T1 LMX-C with T2 LMX-C, and T1 AOC with T3 AOC	1774.96*	676	.87	.86	.06	401.96*	13
9. Six-factor solution, combining all self-concept variables	2402.76*	676	.80	.78	.08	1029.75*	13
10. One-factor solution, combining all variables	6102.10*	694	.36	.32	.13	4729.10*	31

*N* = 449, based on full information maximum likelihood estimation. *df*, degrees of freedom; *CFI*, comparative fit index; *TLI*, Tucker-Lewis index; *RMSEA*, root mean square error of approximation; *T1*, time 1; *T2*, time 2; *T3*, time 3; *AOC*, affective organizational commitment; *LMX-C*, leader-member exchange, contribution dimension; *RSC*, relational self-concept; *CSC*, collective self-concept; *ISC*, individual self-concept

*p < .01

### Measurement Invariance

Because our theoretical model controlled for time 1 LMX-Contribution and AOC, we needed to establish that their measurement was invariant across time to ensure that the construct meaning remained stable (Cole & Maxwell, 2003; Millsap, 2011). A sequential approach was adopted (e.g., Vandenberg & Lance, 2000) where increasingly stringent constraints were added to the CFA model of LMX-Contribution and AOC. Robust maximum likelihood (MLR) was used to test measurement invariance. The results are shown in Table 2. As we proceeded to test the sequence of constraints from configural invariance, to weak invariance (i.e., loadings), strong invariance (i.e., loadings and thresholds), and strict invariance (i.e., loadings, thresholds, and uniquenesses), the Satorra-Bentler scaled *χ*^2^ values were nonsignificant at each step for both LMX-Contribution and AOC.^1^ This finding indicates strict invariance for both variables across time, stable psychometric properties, and suitability for longitudinal analysis (Byrne, Shavelson, & Muthén, 1989; Cheung & Lau, 2012). Thus, these specifications were added to the longitudinal tests of our hypotheses.

**Table 2:** Tests of measurement invariance across time

	*χ*^*2*^	*df*	CFI	TLI	RMSEA	Model comparison	*SB* Δ*χ*^*2*^	Δ*df*
LMX-Contribution								
Model 1: Configural invariance	9.89	6	.99	.98	.04	–		–
Model 2: Weak invariance (loadings)	11.18	8	1.00	.99	.03	2 vs. 1	1.27	2
Model 3: Strong invariance (loadings, thresholds)	11.61	10	1.00	1.01	.02	3 vs. 2	0.24	2
Model 4: Strict invariance (loadings, thresholds, uniquenesses)	14.89	13	1.00	1.01	.02	4 vs. 3	3.29	3
AOC								
Model 1: Configural invariance	186.76*	47	.94	.92	.08	–		–
Model 2: Weak invariance (loadings)	196.96*	52	.94	.92	.08	2 vs. 1	4.94	5
Model 3: Strong invariance (loadings, thresholds)	205.70*	57	.94	.93	.08	3 vs. 2	7.86	5
Model 4: Strict invariance (loadings, thresholds, uniquenesses)	206.70*	63	.94	.94	.07	4 vs. 3	2.01	6

Full information maximum likelihood estimation was used. *df*, degrees of freedom; *CFI*, comparative fit index; *TLI*, Tucker-Lewis index; *RMSEA*, root mean square error of approximation; *SB*, Santorra-Bentler scaled

*p < .05

### Descriptive Statistics and Correlations

Descriptive statistics, correlations and reliability coefficients are reported in Table 3. Laissez-faire leadership was negatively related to time 2 LMX (*r* = − .22, *p* < .01) but unrelated to time 3 AOC (*r* = − .15, *ns*). Time 2 LMX-Contribution was positively related to time 3 AOC (*r* = .36, *p* < .01). The relational self-concept was unrelated to laissez-faire leadership (*r* = − .04, *ns*) and time 2 LMX-Contribution (*r* = .04, *ns*) but positively correlated with time 3 AOC (*r* = .18, *p* < .05).

**Table 3:** Descriptive statistics and correlations for the study variables

Variable	*M*	*SD*	1	2	3	4	5	6	7	8	9	10	11	12	13	14	15	16	17	18
1. Age	37.67	9.00	–																	
2. Gender	1.53	0.50	− .14**	–																
3. Organizational tenure (years)	6.07	5.67	.42**	− .12*	–															
4. Tenure with the supervisor (years)	2.95	2.28	.22**	− .10*	.34**	–														
5. Laissez-faire leadership (T1)	2.36	1.11	.10*	.07	.09	.08	(.93)													
6. Relational self-concept (T1)	4.46	0.52	.03	.12*	− .03	.02	− .04	(.71)												
7. Individual self-concept (T1)	2.91	0.92	− .23**	− .14**	− .06	− .03	.10*	− .03	(.82)											
8. Collective self-concept (T1)	4.18	0.62	.14**	.04	.00	.04	.01	.27**	.05	(.77)										
9. LMX-Contribution (T1)	3.78	0.86	.02	− .07	.05	.13**	− .23**	.16**	.08	.34**	(.79)									
10. LMX-Affect (T1)	3.50	1.07	− .02	.01	− .01	− .05	− .50**	.09	.00	.09	.41**	(.90)								
11. LMX-Loyalty (T1)	3.53	1.07	− .10*	− .03	− .03	.00	− .58**	.06	.03	.06	.40**	.68**	(.91)							
12. LMX-Professional respect (T1)	3.47	1.17	− .09	− .01	− .08	.01	− .49**	.07	.06	.16**	.50**	.57**	.54**	(.94)						
13. AOC (T1)	3.23	1.03	.06	− .08	.15**	.09	− .16**	.09	.04	.40**	.37**	.22**	.22**	.30**	(.93)					
14. LMX-Contribution (T2)	3.55	0.91	.07	− .21**	.08	.16*	− .22**	.04	.07	.10	.60**	.32**	.23**	.40**	.23**	(.80)				
15. LMX-Affect (T2)	3.49	1.12	− .08	.05	− .07	− .04	− .45**	.00	.00	.04	.40**	.78**	.57**	.51**	.15*	.43**	(.91)			
16. LMX-Loyalty (T2)	3.44	1.11	− .14	.04	− .04	.01	− .49**	.02	.04	− .05	.27**	.57**	.72**	.40**	.10	.32**	.71**	(.90)		
17. LMX-Professional respect (T2)	3.39	1.19	.00	− .04	− .12	.03	− .47**	− .01	.04	.06	.41**	.45**	.39**	.76**	.16*	.46**	.56**	.51**	(.95)	
18. AOC (T3)	3.19	0.99	− .12	− .02	.21*	.08	− .15	.18*	− .06	.25**	.23*	.13	.14	.32**	.73**	.36**	.17	.24**	.21*	(.93)

Correlations are based on the data available at a given time: T1 *N* = 449, T2 *N* = 182, T3 *N* = 120. For gender, 1 = male, 2 = female. *T1*, time 1; *T2*, time 2; *T3*, time 3; *LMX*, leader-member exchange; *AOC*, affective organizational commitment. Cronbach’s alphas are reported in parentheses along the diagonal

*p < .05; **p < .01

### Hypothesis Testing

We tested our hypotheses through latent moderated structural equation modeling (LMS; Klein & Moosbrugger, 2000; Maslowsky, Jager, & Hemken, 2015; Sardeshmukh & Vandenberg, 2017) and maximum likelihood (i.e., FIML) estimation using numerical integration and raw data. We used the XWITH command in Mplus and robust standard errors estimation. By considering the measurement errors of the observed variables and factoring in the nonnormally distributed interactions of the latent variables, the LMS approach generates reliable estimates and unbiased standard errors, and has increased power to detect interaction effects (Cheung & Lau, 2017; Klein & Moosbrugger, 2000; Sardeshmukh & Vandenberg, 2017). Thus far, LMS is the most efficient and unbiased approach to testing interactions among latent variables (Klein & Moosbrugger, 2000; Sardeshmukh & Vandenberg, 2017; Schermelleh-Engel, Werner, Klein, & Moosbrugger, 2010).

As LMS does not assume multivariate normality, commonly used fit indices (e.g., RMSEA, CFI, TLI; Maslowsky et al., 2015) are not provided. We therefore followed the recommended two-step approach (Dimitruk, Schermelleh-Engel, Kelava, & Moosbrugger, 2007; Sardeshmukh & Vandenberg, 2017) to test our hypotheses. We first assessed the fit of a baseline model where the interaction between laissez-faire and the relational self-concept was constrained to zero. We then compared this model with a model including the interaction term. The two models were compared using a log-likelihood difference test (D-2LL; Dimitruk et al., 2007) and the Akaike information criterion (AIC) and Bayesian information criterion (BIC) indices (Sardeshmukh & Vandenberg, 2017). A significant D-2LL value indicates that the augmented model should be retained as the best model (Dimitruk et al., 2007), while smaller values for the AIC and BIC are needed to ensure that there is no dramatic loss of information relative to the baseline model (Sardeshmukh & Vandenberg, 2017). We used 95% confidence intervals (CIs) from 5000 bootstrap samples (MacKinnon, Lockwood, & Williams, 2004) in Mplus and the ML estimator for testing the significance of the moderation and moderated mediation effects predicted in Hypotheses 1–2.

**Fig. 1:**
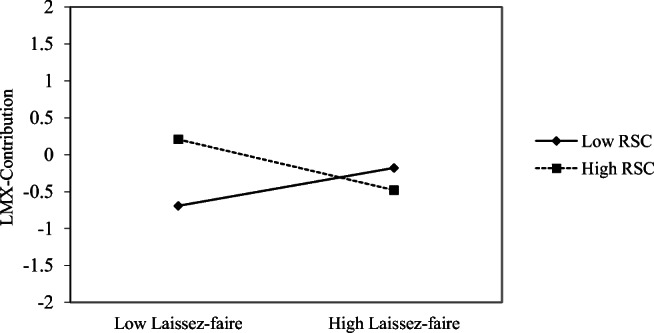
Interaction between laissez-faire leadership and relational self-concept (RSC) predicting LMX-Contribution. Relationships are shown at one 1 *SD* below and above the mean of RSC

**Hypothesis 1.** The baseline model including the main effects of laissez-faire leadership and relational self-concept on time 2 LMX-Contribution, controlling for time 1 LMX-Contribution and the main effects of individual and collective self-concepts, yielded a good fit to the data (*χ*^2^(311) = 636.83, CFI = .93, TLI = .92, RMSEA = .05). However, the moderated model outperformed the baseline model (D-2LL(1) = 10.29, *p* < .01). Moreover, this model displayed smaller values for the AIC (27,594.32 vs. 27,601.25) and BIC (27,984.48 vs. 27,987.31). Thus, the moderated model was retained. As shown in Table 4, the interaction between laissez-faire leadership and the relational self-concept predicting LMX-Contribution was significant (*B* = − .67, *SE* = .28, *p* < .05). The interaction is graphed in Fig. 1. Laissez-faire leadership was significantly negatively related to LMX-Contribution (*B* = − .34, *SE* = .15, *p* < .05) when relational self-concept was high (1 *SD* above the mean) but unrelated to LMX-Contribution (*B* = .26, *SE* = .14, *ns*) when relational self-concept was low (1 *SD* below the mean). Moreover, the difference between these two relationships was significant (*B* = − .60, *SE* = .25, *p* < .05). Interestingly, the relationship between laissez-faire leadership and LMX-Contribution was significantly negative (*p* < .05) when relational self-concept had a standardized value of at least .245 but was significantly positive (*p* < .05) when relational self-concept had a standardized value of − .572 or lower. Hypothesis 1 is thus supported.

**Table 4:** Path analysis results for the moderation and moderated mediation models

	Moderation				Moderated mediation				
	Baseline model		Moderated model		Baseline model		Moderated mediation model		
Variable	*B*	*SE*	*B*	*SE*	*B*	*SE*	*B*	*SE*	95% CI
T1 Laissez-faire → T2 LMX-C	− .06	.09	− .04	.08	− .07	.09	− .05	.08	[− .209, .110]
T1 RSC → T2 LMX-C	.27	.22	.33	.24	.27	.23	.36	.25	[− .151, .885]
T1 CSC → T2 LMX-C	− .29	.16	− .31	.16	− .28	.16	− .31	.17	[− .646, .098]
T1 ISC → T2 LMX-C	− .01	.12	− .02	.12	− .00	.12	− .01	.11	[− .243, .219]
T1 LMX-C → T2 LMX-C	.73***	.09	.74***	.09	.73***	.09	.74***	.09	[.571, .959]
T1 Laissez-faire × T1 RSC → T2 LMX-C			− .67*	.28			− .69*	.28	[− 1.317, − .174]
T1 Laissez-faire → T3 AOC					− .09	.07	− .09	.07	[− .233, .041]
T1 RSC → T3 AOC					.51*	.24	.57*	.25	[.046, 1.122]
T1 CSC → T3 AOC					− .22	.13	− .24	.14	[− .509, .106]
T1 ISC → T3 AOC					− .12	.09	− .12	.09	[− .316, .061]
T1 AOC → T3 AOC					.72***	.06	.72***	.06	[.595, .859]
T2 LMX-C → T3 AOC					.15**	.05	.14**	.05	[.023, .249]
First stage moderation:									
High RSC (+ 1 *SD*)			− .34*	.15			− .36*	.15	[− .672, − .067]
Mean (0)			− .04	.08			− .05	.08	[− .209, .110]
Low RSC (− 1 *SD*)			.26	.14			.26	.15	[− .015, .595]
Difference (± 1 *SD*)			− .60*	.25			− .62*	.25	[− 1.185, − .156]
Indirect effect									
High RSC (+ 1 *SD*)							− .05*	.03	[− .111, − .002]
Mean (0)							− .01	.01	[− .033, .017]
Low RSC (− 1 *SD*)							.04	.02	[− .004, .098]
Difference (± 1 *SD*)							− .09*	.05	[− .197, − .007]

*N* = 449, based on full information maximum likelihood estimation. *B*, unstandardized beta coefficient; *SE*, standard error; *CI*, confidence interval; *T1*, time 1; *T2*, time 2; *T3*, time 3; *LMX-C*, leader-member exchange, contribution dimension; *RSC*, relational self-concept; *CSC*, collective self-concept; *ISC*, individual self-concept; *AOC*, affective organizational commitment

*p < .05; **p < .01; ***p < .001

**Hypothesis 2.** The moderated mediation relationship predicted in Hypothesis 2 was tested following Sardeshmukh and Vandenberg’s (2017) recommendations. We first specified a mediation model including (a) the main effects of laissez-faire leadership and relational self-concept on time 2 LMX-Contribution, controlling for time 1 LMX-Contribution, and on time 3 AOC, controlling for time 1 AOC, and (b) the effect of time 2 LMX-Contribution on time 3 AOC. Moreover, the model controlled for the main effects of the individual and collective self-concepts on time 2 LMX-Contribution and time 3 AOC. This baseline model showed an acceptable fit (*χ*^2^(688) = 1328.24, CFI = .92, TLI = .91, RMSEA = .05). We then compared this model with a moderated mediation model in which relational self-identity moderated the first stage of the mediated relationship between laissez-faire leadership and time 3 AOC through time 2 LMX-Contribution. The latter model outperformed the baseline model (D-2LL(1) = 9.31, *p* < .01) and displayed smaller values for the AIC (35,619.84 vs. 35,627.67) and BIC (36,161.97 vs. 36,165.69). Thus, this model was retained and used to examine the conditional indirect effects of interest.

**Fig. 2:**
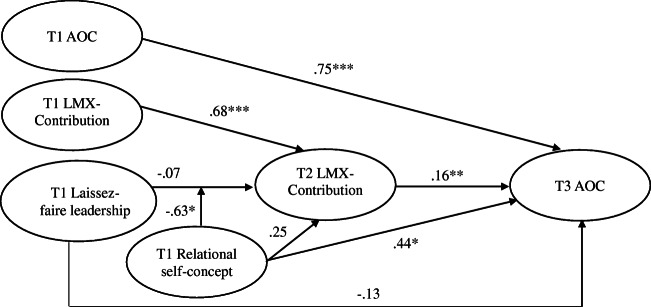
Standardized parameter estimates for the moderated mediation model. T1, time 1; T2, time 2; T3, t3; LMX, leader-member exchange; AOC, affective organizational commitment. For the sake of parsimony, control variables (i.e., individual and collective self-concepts) are omitted (their effects are reported in Table 4). Correlations among exogenous variables are not shown. *p < .05; **p < .01; ***p < .001

Using bootstrapping, the indirect relationship between laissez-faire leadership and time 3 AOC through time 2 LMX-Contribution was found to be significantly negative (*B* = − .05, *SE* = .03, 95% CI [− .111, − .002]) when relational self-concept was high (1 *SD* above the mean) but nonsignificant (*B* = .04, *SE* = .02, 95% CI [− .004, .098]) when relational self-concept was low (1 *SD* below the mean) (Table 4). Moreover, the difference between these two relationships was significant (*B* = − .09, *SE* = .05, 95% CI [− .197, − .007]). Notably, the conditional indirect effect of laissez-faire leadership was significantly negative (*p* < .05) when relational self-concept had a standardized value of at least .387. Hypothesis 2 is thus supported. The path estimates associated with the moderated mediation model as obtained by standardizing the data before analysis (e.g., Klein & Moosbrugger, 2000; Maslowsky et al., 2015) are reported in Fig. 2.^2^

### Additional Analyses

We explored the possibility that a relational self-concept could moderate the indirect relationship between laissez-faire leadership and time 3 AOC through the other dimensions of (time 2) LMX, namely affect, loyalty, and professional respect. Using the same procedure as for testing Hypothesis 2, we found the baseline models to display a good fit (LMX-Affect: *χ*^2^(688) = 1196.57, CFI = .94, TLI = .93, RMSEA = .04; LMX-Loyalty: *χ*^2^(688) = 1167.63, CFI = .94, TLI = .94, RMSEA = .04; LMX-Professional respect: *χ*^2^(688) = 1210.72, CFI = .94, TLI = .93, RMSEA = .04). However, the moderated mediation model with time 2 LMX-Affect, LMX-Loyalty, and LMX-Professional respect as alternative mediators did not improve over the baseline model (D-2LL(1) = 2.61, *ns*; D-2LL(1) = 3.30, *ns*; and D-2LL(1) = 1.65, *ns*; respectively). This finding indicates that the relational self-concept did not moderate the indirect relationship between laissez-faire leadership and time 3 AOC through the other dimensions of time 2 LMX.

Similarly, we examined whether the collective and individual self-concepts exerted a similar moderating effect in our mediation model. The baseline model (which was identical in both cases) displayed a good fit (*χ*^2^(688) = 1328.24, CFI = .92, TLI = .91, RMSEA = .05). Unexpectedly, for both self-concept levels, we found that the moderated mediation model improved over the baseline model (D-2LL(1) = 5.75, *p* < .05 (collective self-concept); and D-2LL(1) = 5.77, *p* < .05 (individual self-concept)). In these models, the interaction between laissez-faire and the collective (*B* = − .40, *SE* = .17, *p* < .05) and the individual (*B* = − .29, *SE* = .13, *p* < .05) self-concept were significant predictors of LMX-Contribution. The relationship between laissez-faire leadership and LMX-Contribution was significantly negative at high levels (i.e., 1 *SD* above the mean) of the collective (*B* = − .32, *SE* = .13, *p* < .05) and individual (*B* = − .26, *SE* = .11, *p* < .05) self-concept but nonsignificant at low levels (1 *SD* below the mean) of these moderators (*B* = .19, *SE* = .15, *ns*; and *B* = .10, *SE* = .12, *ns*; respectively). Differences between the two relationships were also significant for both the collective and the individual self-concept (*B* = − .52, *SE* = .22, *p* < .05; and *B* = − .36, *SE* = .16, *p* < .05, respectively).

Moreover, the indirect effect of laissez-faire leadership on AOC through LMX-Contribution was significantly negative (*B* = − .05, *SE* = .02, 95% CI [− .113, − .006]) when collective self-concept was high (1 *SD* above the mean) but nonsignificant (*B* = .03, *SE* = .02, 95% CI [− .004, .108]) when it was low (1 *SD* below the mean); the difference between the two effects was significant (*B* = − .08, *SE* = .04, 95% CI [− .208, − .013]). In contrast, the indirect effect of laissez-faire leadership on AOC was nonsignificant at both high (*B* = − .04, *SE* = .02, 95% CI [− .083, .000]) and low (*B* = .02, *SE* = .02, 95% CI [− .022, .055]) levels of individual self-concept and did not differ across levels of this moderator (*B* = − .05, *SE* = .03, 95% CI [− .116, .000]). We elaborate on these results in the discussion.

## Discussion

This study demonstrates that the relational self-concept acts as an important individual difference variable that affects the strength of the relationships among laissez-faire leadership, the LMX contribution dimension, and AOC. Using a three-wave longitudinal study, these relationships were found to be stronger and negative among employees with strong relational self-concepts. As such, this study is a preliminary attempt to examine the mechanisms and boundary conditions that explain how laissez-faire leadership practices affect subordinates’ reactions. Our conclusions are particularly robust given the use of a longitudinal approach that controlled for the baseline levels of the mediator and outcome variables. The next sections outline the implications of this study for our understanding of laissez-faire leadership.

### Theoretical Implications

The overriding goal of the present study was foremost to address the theoretical gap surrounding the mechanisms and boundary conditions specifying when and how laissez-faire leadership is expected to relate to AOC. This research endeavor was timely given recent calls to increase our understanding of the effects of laissez-faire leadership (Bass & Bass, 2008; Wong & Giessner, 2018; Yang, 2015) and the need to account for subordinates’ characteristics in examining these effects (Nielsen, Skogstad, Gjerstad, & Einarsen, 2019). Building on the identity orientation framework (Brewer & Gardner, 1996), we posited that a relational self-concept drives an employee’s perception and evaluation of the appropriateness of laissez-faire leadership behaviors. Specifically, because dyadic relationships with significant others (e.g., supervisors) are an important part of an employee’s self-definition, employees with strong relational self-concepts have high expectations about their leaders’ behavior. Laissez-faire leadership violates these expectations, resulting in a reduced willingness of employees to contribute to the mutual goals associated with the relationship. As such, this study furthers our knowledge of the role of employees’ characteristics, which are usually neglected in studies about negative leadership (Schyns & Schilling, 2013). It also contributes to a growing body of literature that has highlighted the role of the employee self-concept as an important trait-like variable to consider in work settings (e.g., van Knippenberg et al., 2004).

The present results are consistent with the view that, even if laissez-faire leadership is a form of passive leadership, it can have destructive effects (e.g., Skogstad et al., 2007) because it can damage the employee-supervisor relationship and organizational commitment, at least when employees have strong relational self-concepts. This view extends the LMX literature, which has traditionally focused on the outcomes rather than on the predictors of LMX (Erdogan & Liden, 2002; Yukl, O’Donnell, & Taber, 2009). Furthermore, our results demonstrate that different styles of leadership may foster different aspects of LMX (e.g., Lee, 2005) and provide further support to the benefits of considering a disaggregated approach to the study of LMX. Moreover, previous research has mostly investigated leadership antecedents that may foster LMX, such as transformational leadership (e.g., Wang, Law, Hackett, Wang, & Chen, 2005), neglecting those leadership styles that act as negative antecedents of LMX. The present results suggest that LMX is affected by negative forms of leadership, which should encourage researchers to examine negative reciprocity as a specific mechanism accounting for the sensitivity of LMX to negative leadership.

Nonetheless, the present findings suggest that the negative effects of laissez-faire leadership are not universal. Rather, these effects particularly occur when employees hold strong relational self-concepts. As such, the relational self-concept is particularly important to explain the impact of laissez-faire leadership on AOC, possibly because individuals with relational self-concepts are more inclined to direct their affective reactions toward their exchange partners (Flynn, 2005), which are then generalized to the organization. Consequently, it appears important to consider the intraindividual context of laissez-faire leadership. Our results also echo Johnson and Chang’s (2008) proposition that individual differences may calibrate employees’ relative sensitivity to the antecedents of AOC. The present findings indicate that employees with low relational self-concepts do not reduce their contribution to mutual goals when they are exposed to laissez-faire leadership. They may even increase this contribution when they hold very low relational self-concepts. Thus, laissez-faire leadership cannot be said to be universally detrimental to employees’ relationship with supervisors and attachment to the organization. This observation goes against the literature that has concluded to consistent negative effects of laissez-faire leadership across situations and contexts (e.g., Bass & Bass, 2008).

As self-concepts and their associated needs shape the perception and interpretation of what constitutes appropriate leader behavior, it is actually the congruence between leader behavior and employees’ expectations and needs that would drive employee reactions (e.g., Wong & Giessner, 2018). Thus, leaders need to adjust their behavior to followers’ characteristics, an argument set forth by the theories of situational or contingent leadership (Fiedler, 2006; Vroom & Jago, 2007; Yukl, 2010). By extension, one may think that followers differ in their needs for leadership and that it is the nonresponse to employees’ specific needs that has the largest influence (de Vries, Roe, & Taillieu, 2002). In sum, this study provides a preliminary answer to Bass and Bass’s (2008, p. 1193) call for addressing the question of “when is laissez-faire leadership appropriate and effective?”

### Directions for Future Research

Unexpectedly, all three levels of the self-concept were found to enhance the impact of laissez-faire leadership. Therefore, in addition to the relational expectations associated with the relational self-concept, other mechanisms may come into play. One potential mechanism is that individuals may be sensitive to any threat to their self-definitions and the accomplishment of the goals they are striving for (e.g., Leavitt & Sluss, 2015). Laissez-faire leaders would have negative effects because they would fall short of meeting the expectations and goals associated with all three self-concept levels. We speculate that when any level of the self-concept is high, a feeling of identity threat will emerge from exposure to laissez-faire leadership. For example, as employees with strong individual self-concepts are committed to achieve career goals (Johnson, Chang, & Yang, 2010), they may be frustrated by laissez-faire leaders because they do not take actions that facilitate their career progress. Similarly, employees with strong collective self-concepts take the well-being of their workgroup to heart (Johnson et al., 2010) and may thus be disappointed to see laissez-faire leaders not working at building cohesion within their workgroup, which would threaten their identities as members of the group. This may reduce their contributions to mutual goals and ultimately AOC. In line with these avenues for future inquiry, past research has suggested that the same leadership style may influence multiple identity-related processes among employees (e.g., Wu et al., 2010). Future research is needed to examine how laissez-faire leadership can threaten the achievement of the goals associated with each of the self-concept levels.

Another avenue for future research would be to examine why leaders engage in laissez-faire behaviors. Do they simply engage in laissez-faire behaviors unknowingly or because they do not have the desire, the knowledge, or the resources to fulfill their prescribed role? Courtright, Colbert, and Choi (2014) suggested that leaders may engage in such behaviors due to developmental challenges and emotional exhaustion. Studying the antecedents of and potential explanations for such behaviors would increase our knowledge regarding when laissez-faire leadership behaviors emerge in the workplace, hence contributing to leadership development (Day, Fleenor, Atwater, Sturm, & McKee, 2014). While these reasons may differ across leaders, identifying those factors that foster laissez-faire practices would help work against its potentially harmful effects and implement interventions that limit their occurrence. For example, examining leaders’ own self-concept levels would be worthwhile (van Knippenberg et al., 2004). Speculatively, leaders with strong individual self-concepts may be more focused on their own ambitions and personal goals, thus neglecting employees’ needs, which may pave the way for laissez-faire behaviors. These leaders may want to move up the corporate ladder and think that a management position is a step toward this goal, even in the absence of a personal desire to supervise employees. Previous research has associated the individual self-concept with more frequent abusive behaviors (Johnson, Venus, Lanaj, Mao, & Chang, 2012). This logic could be extended to laissez-faire leadership, with stronger individual self-concepts making leaders more prone to engage in laissez-faire behaviors.

More generally, laissez-faire leadership remains an understudied form of leadership. One area where more work is needed concerns the similarities and differences between laissez-faire leadership and other destructive forms of leadership. In a recent meta-analysis of destructive leadership in military contexts, Fosse, Skogstad, Einarsen, and Martinussen (2019) found that active-destructive leadership (e.g., abusive supervision, supervisor undermining) and passive-destructive leadership (e.g., laissez-faire) had similar negative relationships with job performance, job attitudes, and employee health and well-being. However, as LMX was not included in the outcomes addressed in this meta-analytic review, it remains unclear how the different forms of destructive leadership distinctively contribute to undermine LMX development and whether some LMX dimensions are particularly affected by them. Future research should thus attempt to disentangle the effects of the different forms of destructive leadership on LMX development.

### Practical Implications

Organizations should train leaders to detect, reduce, and understand the implications of laissez-faire behaviors, just as they do for positive leadership practices. This approach would help practitioners to know not only when to act but also when not to act. Practitioners should be aware that appropriate actions may not only depend on situations per se but on an employee’s specific needs as well. Discrepancies between the leader’s behaviors and the employee’s expectations or specific needs may explain the relative impact of laissez-faire leadership. Therefore, interventions implemented to increase the quality of relationships between employees and leaders and to foster organizational commitment must be adapted based on employees’ levels of the self-concept because these levels drive their expectations. As our research has shown, even individuals who tend to focus on contributing to others’ well-being (i.e., with a strong relational self-concept; Brewer & Gardner, 1996) are still capable of developing attitudes and behaviors that go against their natural tendencies. Thus, laissez-faire leadership may result in the relational potential of employees being wasted because it promotes inappropriate behaviors. Practitioners should take the time to get to know their employees’ needs and self-concepts, communicate on these aspects, and strive to fulfill employees’ expectations. Hence, organizations should pay greater attention to the diversity of employees’ characteristics to fully realize the potential of their employees. Recognizing the diversity of identity-related expectations should be reflected in programs and practices, such as recruitment and socialization processes (Ashforth & Schinoff, 2016), that are tied to employees’ self-concept orientations (Pratt, 2000). By taking advantage of these diverse opportunities, organizations could build stronger bonds and hope for better performance and increased retention among employees.

### Limitations

As study measures were self-reported, common method bias may be an issue (Podsakoff, MacKenzie, & Podsakoff, 2012). Nonetheless, self-reports might be appropriate given our focus on perceptions of self-identity levels and attitudes in the workplace (Conway & Lance, 2010; Spector, 2006). Previous research on the self-concept has indeed traditionally relied on self-report measures (Byrne, 2002). Moreover, our longitudinal analysis controlled for the baseline levels of both the mediator (i.e., LMX-Contribution) and outcome (i.e., AOC) variables, thus considerably reducing any endogeneity related to our findings (Podsakoff, MacKenzie, Lee, & Podsakoff, 2003) and lending confidence to their robustness. Furthermore, because our hypotheses focused on the interaction between laissez-faire leadership and the relational self-concept, common method variance is unlikely to have affected the findings (Siemsen, Roth, & Oliveira, 2010). We also recognize that this study used a specific sample of highly educated employees from a culturally individualistic context. It is possible that different results would be found in a collectivistic culture, as self-concepts are known to be developed in relation to the social context and to vary across cultures (Oyserman, 2001). Hence, people from a Western culture would have stronger individual self-concepts, while those from Eastern countries would possess stronger collective identities (Kitayama, Markus, Matsumoto, & Norasakkunkit, 1997). Nonetheless, even if some findings seem to support a universalist perspective of the self (Sedikides et al., 2011), future research is needed to further examine the generalizability of our findings. Finally, we used the LMX-MDM measure (Liden & Maslyn, 1998) to capture the social exchange-based relationship between employees and leaders. However, this instrument has been criticized for providing an imperfect assessment of social exchange, leading to the development of *leader-member social exchange* (LMSX) as an alternative measure of the construct (Bernerth, Armenakis, Feild, Giles, & Walker, 2007). It would be worth exploring whether the current findings could be replicated using this alternative measure of social exchange relationships in employee-supervisor dyads.

## Conclusion

The present study indicates that laissez-faire leadership negatively relates to AOC through decreased levels of the LMX contribution dimension but only when the employee’s relational self-concept is high. As such, this study highlights how relational expectations can strengthen the (negative) impact of laissez-faire leadership and reveals that it is through employees’ reduced contribution to mutual goals that AOC comes to be affected by laissez-faire leadership. We hope the present study will encourage future attempts at exploring the conditions and mechanisms associated with the effects of laissez-faire leadership in organizations.

## Footnotes

1 Note that we did not allow the errors of T1 item 1 and T2 item 1 to correlate in the invariance tests of the LMX-Contribution scale across time because the models including that specification did not converge.

2 Although our study controlled for the baseline levels of the mediator (LMX-Contribution) and AOC, the data were not cross-lagged, making it possible that AOC drives LMX-Contribution over time. To examine this possibility, we used data from a separate sample to test a cross-lagged model of LMX-Contribution and AOC over a period of 6 months. These data were part of a larger project examining job attitudes and the study was conducted in French. Participants were recruited through convenience sampling from the network of the research team. The time 1 sample comprised 312 respondents. In this initial sample, 22 participants changed organizations and 37 participants changed supervisors before the time 2 survey (i.e., six months later), hence were dropped, reducing the sample to 253 individuals. In this sample, 119 participants provided usable responses at time 2. We first examined whether attrition over time was randomly distributed by conducting a logistic regression analysis predicting the probability of remaining in (vs. being dropped from) the final sample among time 1 respondents (*N* = 253) using time 1 variables (i.e., LMX-Contribution and AOC) as predictors. The logistic regression model was nonsignificant (*χ*^2^(2) = 3.51, *ns*) and none of the predictors was significant. This indicates that data were missing completely at random (MCAR; Ployhart & Vandenberg, 2010) over time, allowing us to examine our cross-lagged model using all the available information from the covariance matrix (*N* = 253) through the FIML method and MLR in Mplus (version 7.31 was used; Muthén & Muthén, 2010). In the sample (*N* = 253), 63% of the participants were female, average age was 38.77 years (*SD* = 10.22), and average organizational tenure was 7.89 years (*SD* = 6.05). Participants worked in a variety of industries such as professional, scientific and technical services (23%), health services and social assistance (18%), and finance, insurance, real estate and public administration (16%). AOC was measured using the same 6-item, adapted version of Meyer et al.’s (1993) original scale (Bentein et al., 2005) as in the main study. The same 3-item scale of LMX-Contribution from the LMX-MDM instrument (Liden & Maslyn, 1998) as in the main study was also used. The internal consistency was good for both AOC (*α*s = .88 and .89 at time 1 and time 2, respectively) and LMX-Contribution (*α*s = .76 and .74 at time 1 and time 2, respectively). The strict invariance model was used for AOC and the weak invariance model was used for LMX-Contribution because adding further constraints revealed significant differences with less constrained models of invariance. Nonetheless, retaining weak invariance still allows testing the relations among latent constructs (Vandenberg & Lance, 2000). The cross-lagged model yielded a good fit to the data (*χ*^2^(141) = 223.77, *p* < .001, CFI = .95, TLI = .95, RMSEA = .05). In this model, time 1 LMX-Contribution was significantly related to time 2 AOC (*B* = .24, *SE* = .09, *p* < .01), controlling for its autoregressive effect (*B* = .67, *SE* = .08, *p* < .001). In contrast, time 1 AOC did not relate to time 2 LMX-Contribution (*B* = .01, *SE* = .10, *ns*), controlling for its autoregressive effect (*B* = .74, *SE* = .13, *p* < .001). These results provide support for the idea that LMX-Contribution temporally precedes AOC, which is consistent with the model presented in Fig. 2.
